# NADPH-dependent peroxidase activity of antibodies in patients with schizophrenia

**DOI:** 10.1192/j.eurpsy.2024.598

**Published:** 2024-08-27

**Authors:** L. Smirnova, D. Kazantseva, E. Kornetova

**Affiliations:** ^1^Laboratory of Molecular Genetics and Biochemistry; ^2^Affective States Department, Mental Health Research Institute Tomsk National Research Medical Center Russian Academy of Sciences, Tomsk, Russian Federation

## Abstract

**Introduction:**

The development of oxidative stress in patients with schizophrenia is associated with changes in the level of activity of antioxidant enzymes. It is likely that catalytically active antibodies (abzymes) can take on these functions. Abzymes are antibodies with enzymatic activity. Catalase and SOD activity of abzymes was previously detected in patients with schizophrenia. But NADPH-dependent peroxidase activity has not been studied. The present work discusses the protective role of abzymes against reactive oxygen species within the pathogenesis of schizophrenia.

**Objectives:**

The aim of the study was to investigate the NADPH-dependent peroxidase activity of IgG in patients with paranoid schizophrenia in the exacerbation phase and in the remission phase.

**Methods:**

A total of 124 patients were examined during the work. Of them, 82 patients with paranoid schizophrenia (F20.0) had a mean age of 33.6±5.12 years (52 males, 30 females), disease duration averaged 8.9± 4.62 years. Patients with schizophrenia included 42 patients with acute schizophrenia and 40 patients with schizophrenia in therapeutic remission. The control group included 42 sex- and age-matched patients. IgG was purified by affinity chromatography on columns with proteinsepharose on an AKTA purifier chromatograph (GE). The homogeneity of isolated IgG preparations was checked by Lemilly electrophoresis in a gradient of 4-18% PAAG. Gel filtration under pH-shock conditions was performed on a Superdex-200 HR 10/30 column. NADPH-dependent peroxidase activity of IgG was determined on a SPECORD M-40 spectrophotometer (Carl Zeiss) at 340 nm by NADPH oxidation in the conjugated glutathione reductase reaction of tertiary butyl hydroperoxide reduction. Statistical processing of data was performed in Statistica 12.0 program.

**Results:**

It was proved that IgG from patients with schizophrenia had NADPH-dependent peroxidase activity, and this activity is an intrinsic property of the investigated antibodies. The NADPH-dependent peroxidase activity in IgG patients in the exacerbation stage was increased 3-fold (p=0.0001) compared to the studied activity in the group of healthy individuals, and it was increased 2-fold (p=0.017) in the group of patients in therapeutic remission compared to the activity in healthy individuals. Also NADPH-dependent IgG peroxidase activity in patients in remission was 1.7 times lower than in patients during the exacerbation period (p=0.012).

**Conclusions:**

It was established for the first time that abzymes from patients with schizophrenia and healthy individuals have NADPH-dependent peroxidase activity and can decompose lipo and hydroperoxides. We hypothesize that these abzymes help cope with generalized oxidative stress. Under the influence of neuroleptic therapy in patients in remission, the level of oxidative stress and NADPH-dependent peroxidase activity of abzymes decrease.

**Disclosure of Interest:**

None Declared

